# Left atrial volume and exercise capacity in adult heart transplant recipients

**DOI:** 10.1186/1749-8090-6-9

**Published:** 2011-01-19

**Authors:** Vitor Oliveira Carvalho

**Affiliations:** 1 Laboratório de Insuficiência Cardíaca e Transplante do Instituto do Coração do Hospital das Clínicas da Faculdade de Medicina da USP (InCor HC-FMUSP), São Paulo, Brazil

## Letter to the editor

It is well established that heart transplantation can improve patients' quality of life, exercise capacity and survival [1,2]. Despite of the improvement in exercise performance after heart transplant, it still remains subnormal when compared with healthy subjects [3]. Cardiac causes as chronotropic incompetence [4] and diastolic dysfunction [5] have been proposed to be related to the post-transplant exercise impairment, but few studies are available about this theme.

The study by Abdul-Waheed et al. [6] is very interesting and adds important information to what we know about cardiac causes of exercise capacity limitations in heart transplant recipients. This retrospective study investigated the left atrial volume (LAV, n = 50) and its change along one year (ΔLAV, n = 40) in transplant recipients.

The main find of the study by Abdul-Waheed et al was the increasing of the LAV along the one year follow-up and the modest correlation between LAV (r = 0.3; p = 0.038) and ΔLAV(r = 0.48; p = 0.002) with VE/VCO_2 _slope, what made the authors speculate about the surgical scar between the native and donor atrium. This scar could impair left atrial pump function and induce atrium dilatation to increase its capacity, as a compensatory mechanism.

In the figure one of the study, it is evident that some patients decreased LAV while the greatest part of patients increased LAV along the follow-up. Maybe if the authors have made the correlation between the ΔLAV and VE/VCO_2 _slope separately, according to the increasing or decreasing of the LAV along the follow-up, it could be figured out that patients who had a decreased LAV could have experienced a negative correlation between ΔLAV and VE/VCO_2 _slope.

This way, these data suggest that LAV may play an important role on the exercise capacity understanding in heart transplant recipients.
